# Antioxidant and Anti-Inflammatory Activities of *Stellera chamaejasme* L. Roots and Aerial Parts Extracts

**DOI:** 10.3390/life13081654

**Published:** 2023-07-29

**Authors:** Temuulen Selenge, Sara F. Vieira, Odontuya Gendaram, Rui L. Reis, Soninkhishig Tsolmon, Enkhtuul Tsendeekhuu, Helena Ferreira, Nuno M. Neves

**Affiliations:** 1 Department of Biotechnology and Nutrition, School of Industrial Technology, Mongolian University of Science and Technology, 8th Khoroo, Baga Toiruu 34, Sukhbaatar District, Ulaanbaatar 14191, Mongolia; temuulen.observe@gmail.com (T.S.); enhtuul_ts@must.edu.mn (E.T.); 2 3B’s Research Group, I3BS—Research Institute on Biomaterials, Biodegradables and Biomimetics, University of Minho, Headquarters of the European Institute of Excellence on Tissue Engineering and Regenerative Medicine, AvePark, Parque de Ciência e Tecnologia, Zona Industrial da Gandra, 4805-017 Guimarães, Portugal; sara.vieira@i3bs.uminho.pt (S.F.V.); rgreis@i3bs.uminho.pt (R.L.R.); helenaferreira@i3bs.uminho.pt (H.F.); 3 ICVS/3B’s–PT Government Associate Laboratory, Braga/Guimarães, Portugal; 4 Department of Pharmaceutical Chemistry and Pharmacognosy, Mongolian University of Pharmaceutical Sciences, Sonsgolon’s Road 4/A Songinokhairkhan District 20th Khoroo, Ulaanbaatar 46520, Mongolia; odontuyag@mas.ac.mn; 5 Tana Lab, Graduate School of Business, Mongolian University of Science and Technology, Sukhbaatar District, Ulaanbaatar 14191, Mongolia; nutrition1healthydiet2@gmail.com

**Keywords:** *Stellera chamaejasme*, chemical fingerprint, antioxidant activity, anti-inflammatory activity, cytocompatibility

## Abstract

Natural products, mainly plants, have a crucial role in folk medicine. Particularly, *Stellera chamaejasme* L. has been traditionally used in Mongolian medicine to treat various diseases, including chronic tracheitis, tuberculosis, and psoriasis. In this study, ethanol (EtOH) and dichloromethane (DCM) extracts of its roots (R) and aerial parts (AP) were evaluated for their antioxidant and anti-inflammatory activities. Thin-layer chromatography demonstrated the presence of flavonoids, namely kaempferol and quercetin-3-O-glucopyranoside, only in the EtOH-AP. Conversely, it showed that kaempferol, quercetin-3-O-glucopyranoside, coumarin, luteolin, rutin, morin, and riboflavin were not present in the other three extracts. The *S. chamaejasme* extracts exhibited strong antioxidant activity. In addition, the roots extracts presented the highest antioxidant activity against peroxyl radicals, with the EtOH-R being the most potent (IC_50_ = 0.90 ± 0.07 µg/mL). *S. chamaejasme* extracts also efficiently inhibited the production of one of the main pro-inflammatory cytokines, interleukin (IL)-6, in a dose-dependent manner by lipopolysaccharide-stimulated macrophages. Particularly, DCM-R was the strongest extract, reducing ≈ 91.5% of the IL-6 production. Since this extract was the most effective, gas chromatography–mass spectrometry (GC-MS) analyses were performed and demonstrated the presence of two fatty acids (palmitic acid and 9-octadecenoic acid), one fatty alcohol (1-hexadecanol), and one triterpenoid (squalene) that can contribute to the observed bioactivity. Herewith, *S. chamaejasme* extracts, mainly DCM-R, exhibit antioxidant and anti-inflammatory activities that could be applied as new and innovative natural formulations for the treatment of chronic inflammatory diseases.

## 1. Introduction

*Stellera chamaejasme* L. is a perennial medicinal plant in the family Thymelaeaceae. Distributed across a wide geographic area, including Russia, Tibet, China, and Mongolia, it is characterized by its white, pink, or yellow flowers [1]. All plant parts, especially roots, have been widely used in both Mongolian and Chinese traditional medicine due to their multiple biological functions, such as anti-inflammatory and anti-cancer activities. Furthermore, it has toxic properties against insect pests, bacteria, and viruses. In addition, it is toxic when eaten by humans and livestock, resulting in diarrhea, vomiting, intestinal decomposition, and possible death [2,3]. Nevertheless, *S. chamaejasme* was recognized as a traditional medicine for treating inflammation by the World Health Organization (WHO) [4].

Inflammation is an intrinsic biological response of the immune system to protect the body against invading pathogens or trauma [5,6]. However, failure to properly regulate the mechanisms that resolve inflammation can lead to the development of uncontrolled and persistent inflammation. In this scenario, large amounts of pro-inflammatory mediators such as free radicals are produced by activated immune cells, including macrophages. Among reactive oxygen and nitrogen species (ROS/RNS), the peroxyl radical (ROO^•^) is one of the most adverse biological free radicals that can react with and impair all types of biological molecules (e.g., lipids, proteins, and DNA) [7]. Consequently, antioxidants are vital substances for protecting our bodies from damage generated from the accumulation of free radicals [8]. Moreover, free radicals can induce some inflammatory pathways, resulting in the production of several pro-inflammatory cytokines, such as tumor necrosis factor-α (TNF-α), interleukin (IL)-1, and IL-6 [9]. An excessive inflammatory response can cause various chronic inflammatory diseases, including osteoarthritis, rheumatoid arthritis, and psoriatic arthritis [10]. Currently, nonsteroidal (e.g., diclofenac and salicylic acid) and steroidal (e.g., dexamethasone) anti-inflammatory drugs, among others, are used for the treatment of inflammation. However, the continuous use of these anti-inflammatory drugs is associated with several and severe adverse effects, including digestive disorders and humoral immune deficiency [6,9]. To avoid these side effects, the search for and discovery of active ingredients from natural sources to develop clinically safe and effective formulations have been increasing [11]. Indeed, around 35 percent of medicines originate from natural products or their derivatives, which comprise one-third of the best-selling drugs globally [12]. 

In the last decades, a large number of *S. chamaejasme* bioactive compounds have been isolated and characterized. The main active compounds are flavonoids, coumarins, diterpenoids, lignans, sesquiterpenes, and volatile oils [13]. In addition, several pharmacological studies demonstrated that *S. chamaejasme* extracts possess a broad range of biological activities, mainly anti-cancer [11,14,15], antibacterial [16], and antioxidant [17] activities. Furthermore, ethanolic extracts of *S. chamaejasme* aerial parts demonstrated cutaneous wound healing in Sprague–Dawley rats and inhibited the release of inflammatory mediators (e.g., TNF-α and IL-1β) in lipopolysaccharides (LPS)-stimulated RAW 264.7 macrophages [4]. The selection of the solvent used in a particular extraction process significantly influences the composition of the obtained extracts [18]. Different solvents have varying polarities that will affect the solubility and extraction yield of specific bioactive constituents. Ethanol, methanol, and water have been extensively used for the preparation of *S. chamaejasme* extracts [4,19,20,21,22]. Conversely, to the best of our knowledge, the use of dichloromethane (DCM) in the extraction of *S. chamaejasme* compounds has not been reported. From our previous work, we demonstrated that this organic solvent recovered more hydrophobic compounds that also exhibited stronger bioactivity [23]. Therefore, in the current study, we investigated the biological properties of DCM extracts obtained from *S. chamaejasme* roots (R) and aerial parts (AP). In order to compare the efficacy of these extracts, ethanol (EtOH) was also employed in the extraction process. Then, after evaluation of their composition by thin-layer chromatography (TLC) and gas chromatography–mass spectrometry (GC-MS), their antioxidant and anti-inflammatory activities were assessed. The antioxidant activity of the extracts was evaluated against different radicals, namely 2,2-diphenyl-1-picrylhydrazyl radical (DPPH^•^), 2,2′-azino-bis (3-ethylbenzothiazoline-6-sulfonic acid), monocation radical (ABTS^•+^), and ROO^•^. DPPH assay was selected since it is considered the easiest and the fastest colorimetric method for low-cost evaluation of the radical scavenging activity of a sample [9]. However, as the same antioxidant molecule may eliminate different free radicals [10], additional scavenging assays were performed. In terms of the ABTS assay, it is one of the most reliable and straightforward assays for antiradical activity assessment. However, DPPH^•^ and ABTS^•+^ are free radicals not found in any biological system, which is a limitation of those assays. Conversely, ROO^•^, as previously stated, is the most relevant radical in the human body [9,11]. The anti-inflammatory potential of the extracts was evaluated by their ability to inhibit IL-6 production by LPS-stimulated human macrophages. In detail, IL-6 is a cytokine that plays a major role in the inflammatory and autoimmune processes associated with different ailments, such as rheumatoid arthritis, diabetes, Castleman’s disease, and cancer [12]. To the best of our knowledge, the current study was the first to compare the biological effects between EtOH and DCM extracts of *S. chamaejasme* roots and aerial parts in the scavenging of different radicals and the inhibition of the production of IL-6 by human macrophages. 

## 2. Materials and Methods

### 2.1. Reagents

Aluminum TLC plates and silica gel coated with fluorescent indicator F254 (20 × 20 cm) were acquired from Merck, Germany. EtOH, DCM, methanol, chloroform, sulfuric acid, vanillin, quercetin-3-O-glucopyranoside, kaempferol, coumarin, luteolin, rutin, morin, riboflavin, N,O-bis(trimethylsilyl)-trifluoroacetamide (BSTFA), natural products-polyethylene glycol (NP/PEG), 2,2-diphenyl-1-picrylhydrazyl (DPPH), 2,2’-azino-bis(3-ethylbenzothiazoline-6-sulfonic acid) (ABTS), potassium phosphate dibasic, potassium phosphate monobasic, trolox, fluorescein sodium salt, 2,2′-azobis (2-methylpropionamidine) dihydrochloride (AAPH), phosphate-buffered saline (PBS), LPS, dexamethasone, diclofenac, bovine serum albumin (BSA), and 12-myristate-13-acetate (PMA) were purchased from Sigma, Portugal. Dimethyl sulfoxide (DMSO) was obtained from VWR, Portugal. Fetal bovine serum (FBS), antibiotic solution, Roswell Park Memorial Institute (RPMI) 1640 medium, and Quant-iT PicoGreen dsDNA Kit were acquired from Thermo Fisher Scientific, Portugal. AlamarBlue was purchased from Bio-Rad, Lisbon, Portugal. Human IL-6 DuoSet enzyme-linked immunosorbent assay (ELISA) and DuoSet ELISA Ancillary Reagent Kit 2 were obtained from R&D Systems, Minneapolis, USA. Ultra-pure water was obtained from a Milli-Q Direct Water Purification System (Milli-Q Direct 16, Millipore, Molsheim, France). Coffee filter paper N4 was acquired from a local supermarket (Braga, Portugal).

### 2.2. S. chamaejasme Extracts Preparation

Dried *S. chamaejasme* (batch number 01-20150829) was purchased from “MONOS” pharmaceutical manufacturer of Mongolia. The roots were washed 2–3 times with ultra-pure water and dried in the dark for a week at room temperature (RT). Roots and aerial parts were ground with a blender (Picadora Clássica 123 A320R1, Moulinex, Lisbon, Portugal) before extraction. Then, 8 ± 0.5 g of each material was separately extracted in EtOH or DCM (2 × 200 mL) under magnetic stirring for 24 h at RT. The solvent was replaced after 12 h. Both extractions using the same solvent were combined, filtered through N4 filter paper, and concentrated in a rotary evaporator. The concentrated solution was transferred to a small glass flask protected from the light, and the solvent was fully evaporated with N_2_ gas. The extraction yield (%) was calculated by dividing the dry weight of the extracts by the initial weight of plant material. Then, the dried extracts were appropriately sealed and stored at −80 °C until further analyses. Thus, four extracts were obtained as DCM or EtOH extracts obtained from roots (DCM-R or EtOH-R) and aerial parts (DCM-AP or EtOH-AP). To ensure the reproducibility of the method, this procedure was repeated three times for each *S. chamaejasme* extract, resulting in three batches of extracts.

### 2.3. Characterization of S. chamaejasme Extracts Composition

#### 2.3.1. TLC Analysis 

TLC analysis was performed according to the method developed by Wagner et al. [24]. All extracts and standards were dissolved with methanol before their application onto the plates. For complete solubilization, DCM extracts were sonicated for 10 min at 45 kHz. Two mobile phases were used. One consisted of chloroform and methanol (95:5, *v*/*v*), and the other was composed of chloroform, methanol, and water (7:3:0.4, *v*/*v*/*v*). After drying, the aluminum TLC plates (SiliaPlate, Merck, Germany) of roots were sprayed with 5% sulfuric acid with 1% vanillin and evaluated under visible light. The TLC plates of aerial parts were sprayed with NP/PEG, and the compounds were evaluated under UV light (365 nm). Then, the retention factors (R_F_) of samples and standards were calculated.

#### 2.3.2. GC-MS Analysis 

A DCM stock solution of 1 mg/mL of DCM-R was prepared. The mixture was sonicated for 40 min until complete dissolution. The GC-MS analysis was performed on an Agilent GC instrument (8890 GC, Santa Clara, CA, USA), combined with a mass spectrometer (5977B GC/MSD, Santa Clara, CA, USA). The chromatographic separation was performed on an UltiMetal column (30 m × 250 μm × 0.1 μm, Agilent GC, Santa Clara, CA, USA). BSTFA was used for hydroxyl-containing compounds derivatization. Helium was used as the carrier gas at a steady flow rate of 2.5 mL/min. The oven temperature was set at 65 °C for 5 min. Then, the temperature was increased at a heating rate of 10 °C/min until reaching 380 °C and held for 8 min. The filtration was carried out using membrane filter (0.22 μm, MF-Millipore TM). The injection volume was 1.0 μL. The GC-MS-acquired data were processed using MZmine 3 software to extract the mass spectral features from the sample raw data. The retention time range was between 4.09 min and 44.49 min. The National Institute of Standards and Technology (NIST) mass spectral library was used to determine the chemical compounds of DCM-R. The relative percentages of DCM-R chemical constituents were calculated by averaging peak area reports.

### 2.4. Antioxidant Activity Evaluation

#### 2.4.1. DPPH^•^ Scavenging Assay

The scavenging capacity of DPPH^•^ by the extracts was evaluated using a procedure already described [25]. The dried ethanolic extracts and DCM-AP were dissolved with EtOH and DMSO at ratios 4:1 *v*/*v* and 1:1 *v*/*v*, respectively. The DCM-R was dissolved with DMSO. The concentration of DPPH^•^ (1.9 mM) was adjusted with EtOH to have an absorbance (abs) of 0.38 ± 0.01 for 180 µL at 515 nm and 25 °C in a microplate reader (Synergy HT Multi-Mode Microplate Reader, Bio-Tek, Winooski, VT, USA). Then, 20 µL of extracts at different concentrations were added in triplicate to each well and mixed with 180 µL of the DPPH solution. The initial abs (t = 0) and at every min until 60 min was measured at 515 nm at 25 °C using the microplate reader previously mentioned. Trolox was used as a positive control. The percentage of the DPPH^•^ at a specific time (t = x) was calculated using Equation (1).

(1) $${DPPH}^{•}\left(\%\right)=\frac{{abs}_{t=x}}{{abs}_{t=0}}\times 100$$

The effective concentration of each extract needed to decrease the DPPH^•^ concentration by 50% (half-maximal inhibitory concentration—IC_50_) was obtained by linear regression analysis of the dose–response curves obtained by plotting extract concentrations (μg/mL) versus antioxidant capacity (%). Lower IC_50_ values indicate a higher capacity of the extracts to neutralize DPPH^•^.

#### 2.4.2. ABTS^•+^ Scavenging Assay

The antioxidant capacity of the extracts to neutralize ABTS^•+^ was measured by a slightly modified method of Re et al. [26]. The extracts solutions were prepared as previously described for the DPPH^•^ assay. ABTS was dissolved in ultra-pure water (7 mM) with potassium persulfate (2.45 mM). Then, the ABTS^•+^ concentration was adjusted with EtOH to obtain an abs of 0.45 ± 0.01 for 180 µL at 734 nm and 30 °C in a microplate reader (Synergy HT Multi-Mode Microplate Reader, Bio-Tek, Winooski, VT, USA). Then, 20 µL of extracts at different concentrations were added in triplicate to each well and mixed with 180 µL of ABTS^•+^ solution. The initial (t = 0) abs and at every 5 min until 20 min was measured at 734 nm at 30 °C, using the same microplate reader previously mentioned. Trolox was used as a positive control. The percentage of ABTS^•+^ at a specific time (t = x) was calculated using Equation (2). The IC_50_ was calculated as previously described.

(2) $${ABTS}^{•+}\left(\%\right)=\frac{{abs}_{t=x}}{{abs}_{t=0}}\times 100$$

#### 2.4.3. Antioxidant Activity against ROO^•^

The antioxidant activity of the extracts against ROO^•^ was determined by using fluorescein as the probe and AAPH as the initiator, according to the method of Lúcio et al. [27]. The extracts and trolox (positive control) were dissolved in a potassium phosphate buffer (pH 7.4). Then, 150 µL of the extracts’ solution at different concentrations were added into a white 96-well microplate. Afterward, 25 µL fluorescein (48 nM) and 25 µL AAPH (15 mM) were added to each well. The fluorescence was immediately measured at an excitation wavelength of 485 nm and an emission wavelength of 528 nm for 3 h at 37 °C in a microplate reader (Synergy HT Multi-Mode Microplate Reader, Bio-Tek, Winooski, VT, USA). The area under the curve (AUC) was used to calculate the antioxidant capacity [28] using Equation (3)

(3) $$Antioxidant\;\;Capacity\left(\%\right)=\frac{{AUC}_{extracts}-{AUC}_{blk}}{{AUC}_{blk}}\times 100$$

where AUC_extracts_ is the AUC obtained for the extracts at different concentrations, and AUC_blk_ is the AUC calculated in the solution without extract (blank). The AUC was calculated by integrating the relative fluorescence curve as a function of the time using GraphPad Prism 6 software. The IC_50_ was calculated as previously described.

### 2.5. Biological Studies

#### 2.5.1. *S. chamaejasme* Extracts Solutions Preparation

Stock extracts solutions for the biological experiments were prepared in DMSO. The extracts stock solutions were 40 mg/mL for EtOH-R, EtOH-AP, and DCM-R and 3 mg/mL for DCM-AP due to its low solubility. Then, the stock solutions of each *S. chamaejasme* extract were serially diluted to obtain the final concentrations of 12.5, 25, 50, 100, and 200 μg/mL for EtOH-R, EtOH-AP, and DCM-R and 1.0, 2.1, 4.1, 8.2, and 16.4 μg/mL for DCM-AP. All stock extract solutions were sterilized with a 0.22 μm syringe filter and stored in aliquots at −80 °C until further experiments. The maximum percentage of DMSO (0.33%) used in the cellular assays did not interfere in the metabolic activity of the cells [9].

#### 2.5.2. Anti-Inflammatory Activity Evaluation

The anti-inflammatory activity of *S. chamaejasme* extracts was evaluated using a human peripheral blood monocyte cell line (THP-1) purchased from American Type Culture Collection and according to the procedure developed by Vieira et al. [9]. The monocytes were maintained in complete RPMI (cRPMI) medium supplemented with 10% of FBS and 1% antibiotic/antimycotic solution at 37 °C under 5% CO_2_ atmosphere. For human THP-1 monocytes differentiation into macrophages [29], the cells (cells/well) were seeded in adherent 24-well culture plates with 100 nM PMA. After 24 h of incubation, non-attached cells were removed by aspiration, and the adherent cells were washed twice with warm cRPMI medium. Afterward, the cells were incubated for 48 h in fresh cRPMI medium to ensure the reversion of monocyte to a phenotype of macrophage. For stimulation, macrophages were further incubated for 2 h with 100 ng/mL of LPS in fresh medium. Without removing the stimulus, *S. chamaejasme* extracts at different concentrations (see Section 2.5.1) were added to the LPS-stimulated macrophages and incubated for 22 h. Dexamethasone and diclofenac were dissolved in ethanol and used at 10 μM in the well as positive controls of compounds with anti-inflammatory activity. As a negative control, cells without stimulation (w/o LPS) were used.

IL-6 production was quantified using an ELISA according to the manufacturer’s instructions. Concisely, a 96-well plate for ELISA was coated overnight with a capture antibody specific to this cytokine. Then, the plate was blocked with 1% bovine serum albumin (BSA) for 1 h. Afterward, 100 μL of the supernatants and standards were added. Two hours later, the detection antibody specific to IL-6 was incubated for another 2 h. After substrate addition and color development, the stop solution was mixed. The abs of each well was immediately read at 450 nm and corrected at 540 nm and 570 nm using a microplate reader (Synergy HT Multi-Mode Microplate Reader, Bio-Tek, Winooski, VT, USA). Between all steps, each well was washed three times with wash buffer (0.05% Tween 20 in PBS, pH 7.3). The IL-6 amount in each sample was calculated from the calibration curve, and the values were normalized considering the DNA concentration. The results are expressed as a percentage (%) related to the positive control (non-treated LPS-stimulated macrophages).

#### 2.5.3. Metabolic Activity

The metabolic activity of macrophages was carried out using the alamarBlue assay according to the instructions of the manufacturer. In living cells, alamarBlue reagent (resazurin) is converted to the fluorescent and colorimetric molecule resorufin. cRPMI medium with 10% (*v*/*v*) alamarBlue was added into each well and incubated for 4 h at 37 °C in a humidified 5% CO_2_ atmosphere. The abs was monitored at 570 nm and 600 nm using a microplate reader (Synergy HT Multi-Mode Microplate Reader, Bio-Tek, Winooski, VT, USA). The results are expressed in percentage related to the positive control (non-treated LPS-stimulated macrophages). 

#### 2.5.4. DNA Quantification

The DNA amount was quantified by the total amount of double-stranded DNA using the Quant-iT PicoGreen dsDNA assay kit according to the instructions of the manufacturer. After metabolic activity assays, macrophages were washed twice with sterile PBS, and 1 mL of ultra-pure water was added to each well for cell membrane disruption. Afterward, the bottom of the wells was scratched, and the suspension was collected in Eppendorfs. Each sample was also sonicated for 15 min for complete lysing of cells. Then, samples and DNA standards were added in triplicate to a 96-well black plate, followed by 71.3 μL of PicoGreen and 100 μL of Tris-ethylenediaminetetraacetic acid (EDTA) buffer. The plate was incubated for 10 min in the dark, and the fluorescence was measured with a microplate reader (Synergy HT Multi-Mode Microplate Reader, Bio-Tek, Winooski, VT, USA) using an excitation wavelength of 480 nm and an emission wavelength of 528 nm. The DNA concentration of each sample was calculated using a standard curve relating DNA concentration and fluorescence intensity. The results are expressed in relative DNA concentrations of the positive control (non-treated LPS-stimulated macrophages). 

### 2.6. Statistical Analysis

The data are expressed as mean ± standard deviation and obtained from three independent experiments. Statistical analysis was performed using GraphPad Prism 6.0 software. Analysis of variance (ANOVA) and Tukey’s multiple comparisons test were used for yield extraction and antioxidant activity data. ANOVA and Dunnett’s multiple comparison method were used for anti-inflammatory activity results. Differences between experimental groups were considered significant with a confidence interval of 99% whenever *p* < 0.01. 

## 3. Results

### 3.1. Extraction Yield 

**Figure 1:**
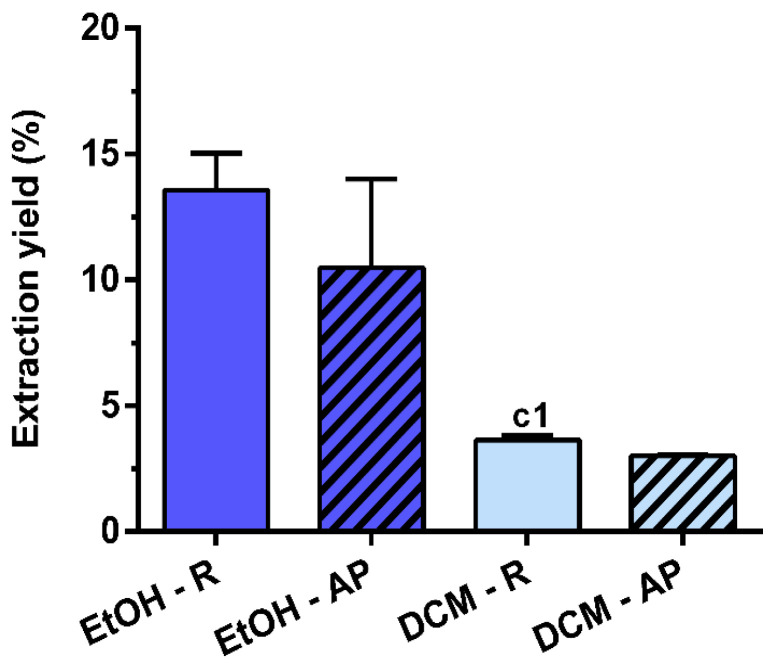
Extraction yield (%) of *S. chamaejasme* extracts. Statistical significance is 1 (*p* < 0.0222) in comparison with c (EtOH-R vs. DCM-R). EtOH, ethanol; DCM, dichloromethane; R, roots; AP, aerial parts.

Different extraction yields were obtained for the four extracts prepared from air-dried *S. chamaejasme* roots and aerial parts under stirring for 24 h with EtOH or DCM (Figure 1). The extraction yield of EtOH preparations was higher because of its greater polarity compared with DCM. Conversely, no differences were observed between the different plant parts that were used. Comparing all the *S. chamaejasme* extracts, EtOH-R (13.6 ± 1.5%) had a similar extraction yield to EtOH-AP (10.5 ± 3.5%), followed by DCM-R (3.6 ± 0.2%) and DCM-AP (3.0 ± 0.1%). 

### 3.2. Chemical Composition of S. chamaejasme Extracts

#### 3.2.1. TLC Analysis of *S. chamaejasme* Extracts

**Figure 2:**
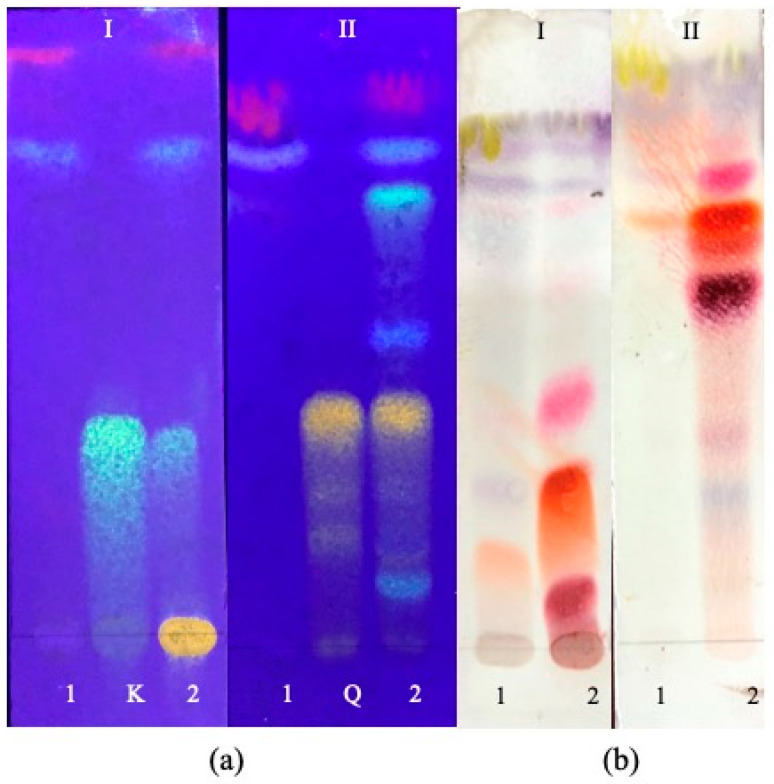
TLC chromatograms of four *S. chamaejasme* extracts obtained with DCM (1) and EtOH (2) from aerial parts (**a**) and roots (**b**) and two standards (K, kaempferol; Q, quercetin-3-O-glucopyranoside). In I and II, the mobile phases used were composed of chloroform and methanol (95:5, *v*/*v*) and chloroform, methanol, and water (7:3:0.4, *v*/*v*/*v*), respectively. Chromatograms were sprayed with NP/PEG (**a**) and revealed using UV (365 nm) or vanillin–sulfuric reagent (**b**) and evaluated in visible light.

According to the TLC analysis of the four *S. chamaejasme* extracts, kaempferol (R_F =_ 0.453 ± 0.013) and quercetin-3-O-glucopyranoside (R_F_ = 0.392 ± 0.005), belonging to flavonoids class, were detected in EtOH-AP (Figure 2). In EtOH-R and DCM extracts, all the tested standards were not observed (Table 1). The TLC chromatograms are shown in Supplementary Materials (Figure 2 and Figures S1–S4). Indeed, due to DCM’s nature, the compounds present in its extracts should be more non-polar. However, in DCM-R and EtOH-R, pink to violet spots were observed, indicating the presence of several compounds. 

#### 3.2.2. GC-MS Analysis of *S. chamaejasme* DCM-R Extracts

**Figure 3:**
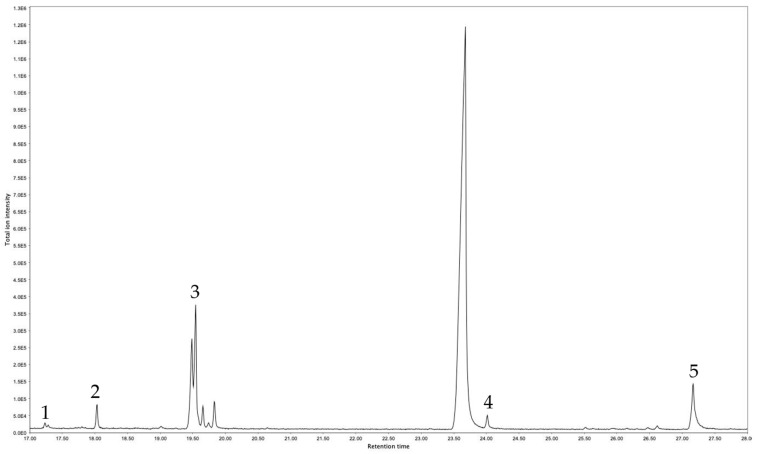
GC-MS chromatogram of *S. chamaejasme* DCM-R.

The GC-MS allows unequivocal identification of the chemical compounds present in *S. chamaejasme* DCM-R extracts. From the ten chromatographic peaks present in DCM-R extract (Figure 3), five chemical compounds were identified (Table 2). DCM-R was composed of two fatty acids (palmitic acid and 9-Octadecenoic acid), one fatty alcohol (1-hexadecanol), and one triterpenoid (squalene). The last identified peak ((Stigmast-5-ene, 3β-(trimethylsiloxy)) corresponds to the derivatized form of beta-sitosterol. 

### 3.3. Extracts Antioxidant Activity

#### 3.3.1. Antiradical Activity of *S. chamaejasme* Extracts against DPPH^•^ and ABTS^•+^

*S. chamaejasme* extracts did not present significant radical scavenging ability against DPPH^•^ and ABTS^• +^ radicals (Figures S6 and S7, respectively). 

#### 3.3.2. Antioxidant Activity of *S. chamaejasme* Extracts against ROO^•^

**Figure 4:**
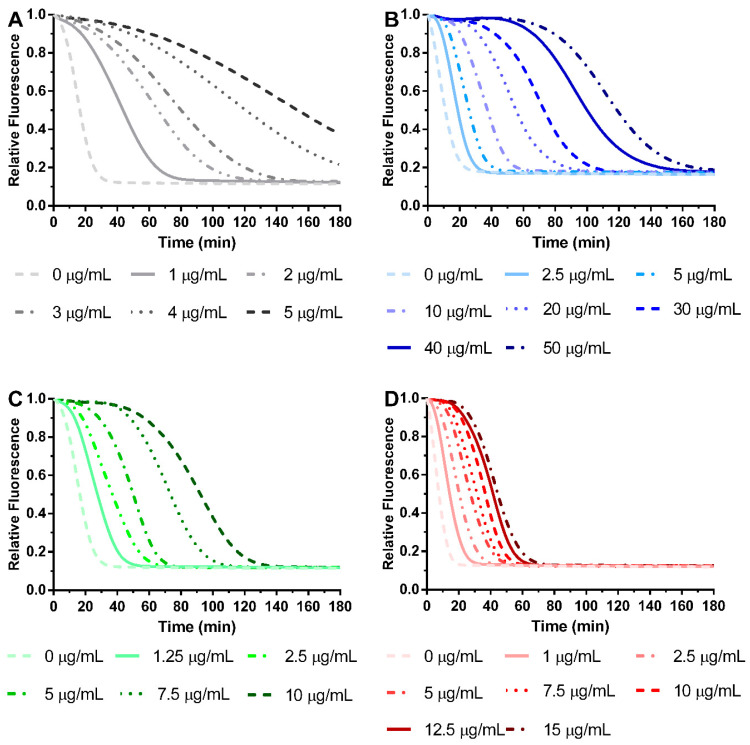
Antioxidant activity of EtOH-R (**A**), EtOH-AP (**B**), DCM-R (**C**), and DCM-AP (**D**) obtained from *S. chamaejasme*, at different concentrations, against ROO^•^. EtOH, ethanol; DCM, dichloromethane; R, roots; AP, aerial parts.

**Figure 5:**
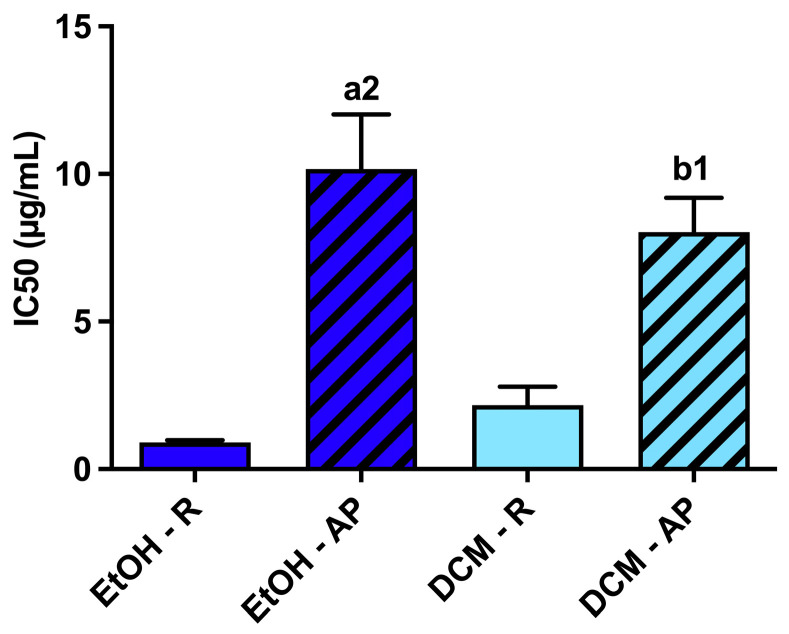
Half-maximal inhibitory concentration (IC_50_, μg/mL) of the different *S. chamaejasme* extracts against ROO^●^. Statistically significant differences are 1 (*p* < 0.0011) and 2 (*p* < 0.0001) in comparison with a (EtOH-R vs. EtOH-AP) and b (DCM-R vs. DCM-AP). EtOH, ethanol; DCM, dichloromethane; R, roots; AP, aerial parts.

All extracts obtained from *S. chamaejasme* presented antioxidant activity against ROO^•^ in a dose-dependent manner (Figure 4), with their IC_50_ (μg/mL) shown in Figure 5. Indeed, they were able to avoid the decay of probe fluorescence [28]. Interestingly, the different solvents did not affect the efficacy of the antioxidant activity of each plant part against ROO^•^. However, significant differences were observed between roots and aerial parts. The root extracts presented the most potent antioxidant activity against ROO^•^. Comparing all the *S. chamaejasme* extracts, EtOH-R (0.90 ± 0.07 μg/mL) and DCM-R (2.17 ± 0.63 μg/mL) presented the most potent antioxidant activity against ROO^•^, followed by DCM-AP (8.03 ± 1.16 μg/mL) and EtOH-AP (10.16 ± 1.86 μg/mL).

### 3.4. Anti-Inflammatory Activity Evaluation

#### 3.4.1. Cytocompatibility of *S. chamaejasme* Extracts

**Figure 6:**
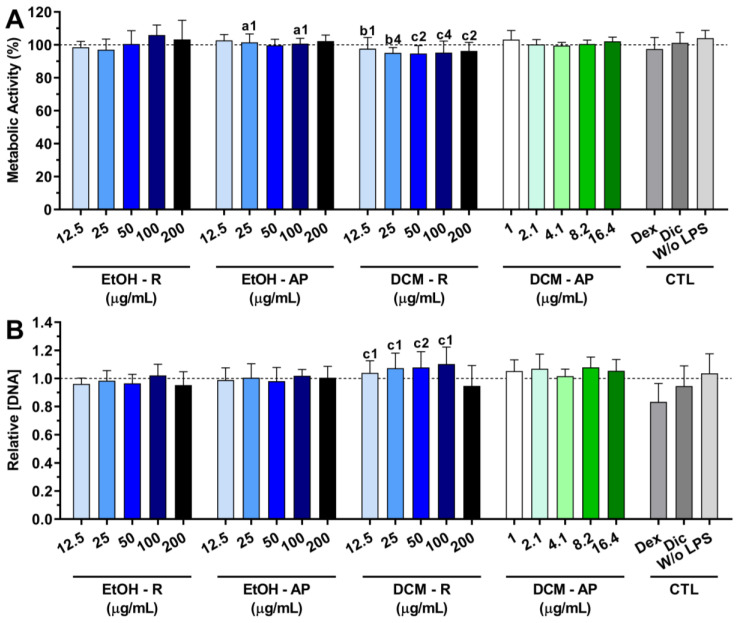
Metabolic activity (**A**) and relative DNA concentration (**B**) of LPS-stimulated macrophages treated with different concentrations of *S. chamaejasme* extracts and clinically used anti-inflammatory drugs (Dex, dexamethasone; Dic, diclofenac—both 10 μM) for 24 h of culture. The dotted line represents the nontreated condition (0 μg/mL) for each assay. There are no statistically significant differences in comparison with positive control (non-treated LPS-stimulated macrophages). Statistically significant differences are 1 (*p* < 0.01), 2 (*p* < 0.001), and 4 (*p* < 0.0001) in comparison with a (EtOH-R vs. EtOH-AP), b (DCM-R—12.5 and 25 µg/mL vs. DCM-AP—16.4 µg/mL), and c (EtOH-R vs. DCM-R). EtOH, ethanol; DCM, dichloromethane; R, roots; AP, aerial parts; CTL, controls.

The cytocompatibility of the *S. chamaejasme* extracts in the presence of LPS-stimulated macrophages is presented in Figure 6. The metabolic activity of macrophages was not affected by the presence of all *S. chamaejasme* extracts (Figure 6A). Moreover, the *S. chamaejasme* extracts at different concentrations preserved the DNA of macrophages under an inflammatory scenario (Figure 6B). A similar profile was obtained for dexamethasone and diclofenac. 

#### 3.4.2. Anti-Inflammatory Activity of *S. chamaejasme* Extracts

**Figure 7:**
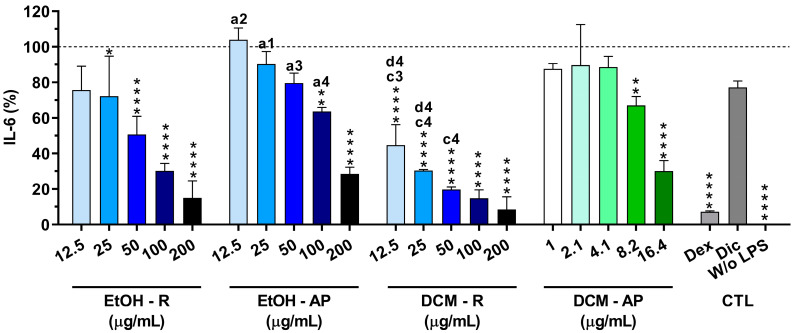
IL-6 amount (%) produced by LPS-stimulated macrophages cultured in the presence of *S. chamaejasme* extracts at different concentrations (12.5, 25, 50, 100, and 200 µg/mL for EtOH-R, EtOH-AP, and DCM-R and 1, 2.1, 4.1, 8.2, and 16.4 µg/mL for DCM-AP) and clinically used anti-inflammatory drugs (Dex, dexamethasone; Dic, diclofenac—both 10 µM) for 24 h. Statistically significant differences are * (*p* < 0.0216), ** (*p* < 0.0093), and **** (*p* ≤ 0.0001) compared to the positive control (non-treated LPS-stimulated macrophages) and 1 (*p* < 0.01), 2 (*p* < 0.001), 3 (*p* < 0.0001), and 4 (*p* < 0.0001) in comparison with a (EtOH-R vs. EtOH-AP), c (EtOH-R vs. DCM-R), and d (EtOH-AP—12.5 and 25 µg/mL—vs. DCM-AP—16.4 µg/mL). EtOH, ethanol; DCM, dichloromethane; R, roots; AP, aerial parts; CTL, controls.

In order to confirm their anti-inflammatory activity, the reduction in the IL-6 production from LPS-stimulated macrophages in the presence of *S. chamaejasme* extracts at different concentrations was investigated (Figure 7). The non-stimulated macrophages (without LPS, 0 µg/mL) did not produce measurable amounts of IL-6. However, macrophages were activated in the presence of LPS (100 ng/mL), leading to a drastic increase in IL-6 production. Dexamethasone (10 μM) dramatically reduced (92.8 ± 0.5%) the IL-6 production. Conversely, diclofenac (10 μM) was not able to significantly diminish the IL-6 production (22.9 ± 3.7%). Interestingly, all *S. chamaejasme* extracts significantly decreased the IL-6 amount in a dose-dependent manner (Figure 7). *S. chamaejasme* extracts obtained with DCM, i.e., both DCM-R and DCM-AP, were more efficient at decreasing the pro-inflammatory cytokine IL-6 production than the extracts prepared with EtOH, as expected. Analyzing the highest tested concentration (200 µg/mL), the DCM-R drastically reduced the IL-6 production from LPS-stimulated macrophages (91.5 ± 7.2%), followed by EtOH-R (84.9 ± 9.5%) and EtOH-AP (71.5 ± 3.8%). In the case of DCM-AP, due to its poor solubility, the highest tested concentration was 16.4 µg/mL, which was able to reduce 69.9 ± 6.0% of the IL-6 production. Analyzing the other three extracts at similar concentrations (between 12.5 and 25 µg/mL), it is possible to observe that DCM-R reduced between 55.4 ± 11.6 to 69.6 ± 0.7% of the IL-6 production, followed by EtOH-R (from 24.3 ± 13.4 to 27.7 ± 22.5%) and EtOH-L (from 0 ± 6.6 to 9.68 ± 7.0%). Therefore, the same concentrations of DCM-AP and DCM-R showed a similar reduction in the IL-6 production.

## 4. Discussion

A large number of plant extracts have shown a tremendous therapeutic potential to treat various illnesses. Indeed, many major currently used drugs are derived from plants, including salicylic acid, codeine, and digitoxin, among others. Moreover, plant-based medicines can possess fewer side effects and be cheaper than synthetic drugs. Consequently, they have been suggested as potential candidates for the prolonged treatment of oxidative stress and inflammation [30,31]. Therefore, in the present study, roots and aerial parts of *S. chamaejasme*, one of the main plants used in traditional Eastern medicine, were used as natural sources of compounds to counteract oxidative stress and inflammation.

It is evident that the extraction solvent dramatically affects not only the efficiency of extraction but also the antioxidant potential [18,32] and the anti-inflammatory activity [32]. Accordingly, in this study, two different organic solvents (EtOH and DCM) with different polarities (EtOH is more polar than DCM) were used to extract bioactive compounds from *S. chamaejasme* to compare the effects of the extraction solvents. Moreover, the influence of plant parts on biological activities was evaluated. 

The extraction yield of *S. chamaejasme* was about 3.5 times higher for EtOH than DCM, indicating that the extraction efficiency depends on the polarity of the solvents. Therefore, its roots and aerial parts contained high levels of more polar constituents that are more soluble in a solvent with high polarity (EtOH). At the same time, no significant differences were observed between roots and aerial parts in relation to yield. 

To evaluate the presence of some compounds in the obtained extracts, TLC analyses were performed. After chromatogram spraying with NP/PEG, some colors indicating flavonoids, including quercetin and kaempferol, were observed in the extracts of the aerial parts of this medicinal plant. According to this observation, we used quercetin-3-O-glucopyranoside and kaempferol as standards and proved that those compounds are present in EtOH-AP (Figure 2). Indeed, there are different studies that demonstrate the presence of quercetin and kaempferol in *Stellera chamaejasme* L. extracts [13,33]. Conversely, and for instance, coumarin was not detected despite many studies proving that especially the roots of this plant contain coumatin derivatives [13,34,35]. Indeed, not only coumarin but also luteolin, rutin, morin, and riboflavin were not detected (Figures S1–S4). Additionally, pink, orange, and brown-red/violet-colored spots were observed boldly in EtOH-R (Figure 2). These spots can be related to compounds with antioxidant properties, including phenylpropane derivatives and terpenes, since they show brown-red/violet and orange color in visible light after treatment with vanillin–sulfuric reagents [36]. However, none of the tested standards were present in the other three extracts. According to the literature, the main chemical compounds extracted from roots of *S. chamaejasme* using EtOH, methanol, or ethyl acetate are flavonoids (e.g., chamaejasmine, isochamaejasmine, quercetin-3-O-glucopyranoside, and rutin) [33,37], coumarins (e.g., daphnoretin, umbelliferone, and 5,7-dihydroxycoumarin) [34], lignans (e.g., pinoresinol, matairesinol, and lirioresinol-B) [38], diterpenoids (e.g., stellerarin and benzoylphorbol) [39], and volatile oils (e.g., caprylic aldehyde and 5-metyl decane) [13]. Moreover, some flavonoids (e.g., isoquercitrin, genkwanin, apigenin, apigenin 7-O-glucoside, luteolin, and taxifolin), coumarins (e.g., daphnin, daphnetin-8-O-glucoside, daphnetin, daphnoretin, and rutarensin), and lignans (e.g., matairesinoside and lariciresinol) were identified and isolated from aerial parts using EtOH [4,21]. Additionally, the total flavonoids of *S. chamaejasme* are present at higher concentrations in the leaves (2.92%) than the roots (1.13%) [16]. Conversely, coumarins are more concentrated in roots (1.87%) compared to leaves (0.33%) [2]. In other words, phenolic compounds, namely phenolic acid, flavonoids, coumarins, lignans, and so forth, are the main class of bioactive phytochemicals correlated with good antioxidant and anti-inflammatory capacities of plants.

An imbalance between free radicals and antioxidants caused by various biological and environmental sources can lead to oxidative stress conditions that can cause inflammation and various other illnesses [40]. In the present study, the antioxidant activity of extracts obtained from *S. chamaejasme* roots and aerial parts was evaluated in the presence of different radicals. Although the extracts did not show significant antiradical activity against DPPH^•^ and ABTS^∙^∙^+^ (Figures S5 and S6), they were able to neutralize ROO^•^ effectively (Figure 4 and Figure 5). This feature is extremely important since this ROS can promote the development of cancer and other diseases related to oxidative stress and ageing [41]. In particular, EtOH-R demonstrated the lowest IC_50_ (0.9 ± 0.07 µg/mL), illustrating that this extract has the most potent antioxidant activity against ROO^•^. Indeed, EtOH can recover more phenolic compounds that are able to efficiently neutralize the radicals. Conversely, DCM extracts contain more hydrophobic compounds with less radical scavenging activity. Thus, the powerful antioxidant activity of roots extracts (EtOH-R and DCM-R) might be related to compounds with more potent antioxidant activity compared to the ones present and identified in the EtOH-AP (quercetin-3-O-glucopyranoside and kaempferol). Consequently, an unequal distribution of antioxidant compounds in the different parts of the plant was observed.

Inflammation is a defense mechanism against infection, irritation, or injury. However, a persistent inflammatory response is associated with various diseases, including cancer and autoimmune diseases [32]. Several stimuli, such as LPS, stimulate macrophages to secrete numerous inflammatory cytokines, such as TNF-α, IL-1, and IL-6 [42]. Among the inflammatory mediators, the pro-inflammatory cytokine IL-6 is one of the main initiators [6]. Several studies reported that the decrease in IL-6 production leads to reduced inflammation in rheumatoid arthritis, asthma, and atherosclerosis [43,44,45]. Results obtained from the investigation of the anti-inflammatory activity showed that the four *S. chamaejasme* extracts efficiently reduced IL-6 production in a concentration-dependent manner (Figure 7). Especially, DCM-R was the most potent anti-inflammatory extract, reducing IL-6 production by 91.5 ± 7.2% at the concentration of 200 μg/mL without significant toxicity to the cells (>90% viability). Additionally, this extract showed similar anti-inflammatory properties as dexamethasone (92.7 ± 0.3%). Due to its low solubility, the highest tested concentration of DCM-AP was 16.4 μg/mL, which inhibited IL-6 production up to 69.9%, making it one potential candidate for inflammation treatment. However, DCM-R was the most promising extract since higher cyto-compatible concentrations could be tested. According to GC-MS, fatty acids, namely palmitic acid and 9-octadecenoic acid, and terpenoid (squalene) as well as one fatty alcohol (1-hexadecanol) were revealed in DCM-R (Table 2). Squalene is not only a powerful natural terpenoid against oxidation but also has strong properties against inflammation that are proven by inhibiting pro-inflammatory cytokines (IL-1 and IL-6) in LPS-mediated murine and human macrophages [46]. Squalene also enhances the levels of anti-inflammatory enzymes. Furthermore, 9-octadecenoic acid demonstrates strong biological properties, including anti-inflammatory, anti-androgenic, and anticancer [47]. Therefore, the chemical composition of the DCM-R is strongly related with its strong antioxidant and anti-inflammatory properties. In addition, according to the literature, the higher activity of DCM extracts, especially from roots, against inflammatory events might be related to the greater solubility of active anti-inflammatory and more hydrophobic compounds, namely coumarins, fatty acids, and diterpenoids. Therefore, the findings from this work assist the research and development of new effective natural medicines for both oxidative stress and inflammation treatment.

## 5. Conclusions

The present study demonstrates that extracts of *S. chamaejasme* roots and aerial parts using EtOH or DCM possess powerful antioxidant and anti-inflammatory activities without significant toxicity. As expected, the four extracts from *S. chamaejasme* had different chemical constituents, as revealed by chemical analysis. Two extracts should be highlighted compared with the others. Firstly, EtOH-R, with moderate anti-inflammatory activity, demonstrated the highest antioxidant activity. Secondly, DCM-R was the most promising extract in inhibiting the pro-inflammatory cytokines and also showed the ability to efficiently neutralize ROO^•^. Taken together, roots are the strongest part of *S. chamaejasme* to be used against both oxidative stress and inflammation. Regarding solvents, DCM was the finest solvent for extracting a complex mixture of compounds with both antioxidant and anti-inflammatory activities, as expected.

To conclude, DCM-R should be further deeply studied since it was the most promising extract of *S. chamaejasme*. The identification of its chemical constituents and the investigation of specific pathways of their mechanisms of action in the reduction in oxidative stress and inflammation are the future developments of our research.

## Figures and Tables

**Table 1:** Presence of the tested standards in the respective extracts of *S. chamaejasme*.

№	Standards	Extracts			
		EtOH-AP	EtOH-R	DCM-AP	DCM-R
1	Kaempferol	+	-	-	-
2	Quercetin-3-O-glucopyranoside	+	-	-	-
3	Coumarin	-	-	-	-
4	Luteolin	-	-	-	-
5	Rutin	-	-	-	-
6	Morin	-	-	-	-
7	Riboflavin	-	-	-	-

“+” and “-” represent the presence and absence of the compound, respectively.

**Table 2:** Chemical compounds present in *S. chamaejasme* DCM-R.

Peak Number	Retention Time (min)	Identified Chemical Compounds	Molecular Formula	Molecular Weight	Peak Area (%)
1	17.235	1-Hexadecanol	C_16_H_34_O	242.4	1.1
2	18.034	Palmitic Acid	C_16_H_32_O_2_	256.4	2.9
3	19.541	9-Octadecenoic acid	C_18_H_34_O_2_	282.5	31.8
4	24.013	Squalene	C_30_H_50_	410.7	1.9
5	27.166	Stigmast-5-ene, 3β-(trimethylsiloxy)	C_32_H_58_OSi	486.9	5.9

## Data Availability

Not applicable.

## Supplementary Materials

The following supporting information can be downloaded at: https://www.mdpi.com/article/10.3390/life13081654/s1, Figure S1: TLC chromatograms of four *S. chamaejasme* extracts obtained with DCM (1) and EtOH (2) from aerial parts (a) and roots (b) and two standards (K: kaempferol, Q: quercetin-3-O-glucopyranoside). In I and II, the mobile phases used were composed of chloroform and methanol (9:1, *v*/*v*) and chloroform, methanol, and water (7:3:0.4, *v*/*v*/*v*), respectively. Chromatograms were sprayed with NP/PEG (*) and revealed using UV (365 nm) or sulfuric acid (+) and vanillin–sulfuric reagent (-) and evaluated in visible light. Figure S2: TLC chromatograms of *S. chamaejasme* extracts including, DCM-AP (1), EtOH-AP (2), DCM-R (3), and EtOH-R (4) and two standards (C1: coumarin from National University of Mongolia, C2: coumarin from MONOS pharmaceutical industry). The mobile phases were chloroform. Chromatogram was evaluated in UV 254 nm (a). Other chromatogram was sprayed with vanillin–sulfuric reagent (b) and evaluated in visible light. Figure S3: TLC chromatograms of two *S. chamaejasme* extracts, including EtOH-AP (1), DCM-AP (2), and DCM-R (3), and four standards (L: luteolin, R: rutin, M: morin, Ri: riboflavin). In I and II, the mobile phases used were composed of chloroform, methanol, and water (7:3:0.4, *v*/*v*/*v*) and dichloromethane, respectively. Chromatograms were sprayed with NP/PEG (a) and revealed using UV (365 nm) or vanillin–sulfuric reagent (b) and evaluated in visible light. Figure S4: TLC chromatograms of *S. chamaejasme* extracts, including DCM-R (1), DCM-R + Quercetin-3-O-glucopyranoside (2), EtOH-R (3), DCM-R (4), EtOH-R+ Luteolin (5), and DCM-R + Luteolin (6), and three standards (K: kaempferol, Q: quercetin-3-O-glucopyranoside, L: luteolin). In I and II, the mobile phases used were composed of chloroform and methanol 9:1 and 6:1 (*v*/*v*), respectively. Chromatograms were sprayed with NP/PEG and revealed using UV (365 nm). Figure S5: Antioxidant activity against DPPH^•^ of varying concentrations of EtOH-AP (A), DCM-AP (B), EtOH-R (C), and DCM-R (D) extracts; Figure S6: Antioxidant activity against ABTS^•+^ of varying concentrations of EtOH-AP (A), DCM-AP (B), EtOH-R (C), and DCM-R (D) extracts.
