# Supplementary material for: Antioxidant and Anti-Inflammatory Activities of Stellera chamaejasme L. Roots and Aerial Parts Extracts
Source: Life (Basel). 2023 Jul 29;13(8):1654. doi: 10.3390/life13081654 (PMC10456005; doi:10.3390/life13081654)
Supplement: Supplementary file 1 [file life-13-01654-s001.zip › life-2145321-supplementary.pdf]

SUPPLEMENTARY MATERIALS to "Antioxidant and anti-inflammatory activities of *Stellera chamaejasme* roots and aerial parts extracts" by Temuulen Selenge , Sara F. Vieira, Odontuya Gendaram, Rui L. Reis, Soninkhishig Tsolmon, Enkhtuul Tsendeekhuu, Helena Ferreira and Nuno M. Neves.

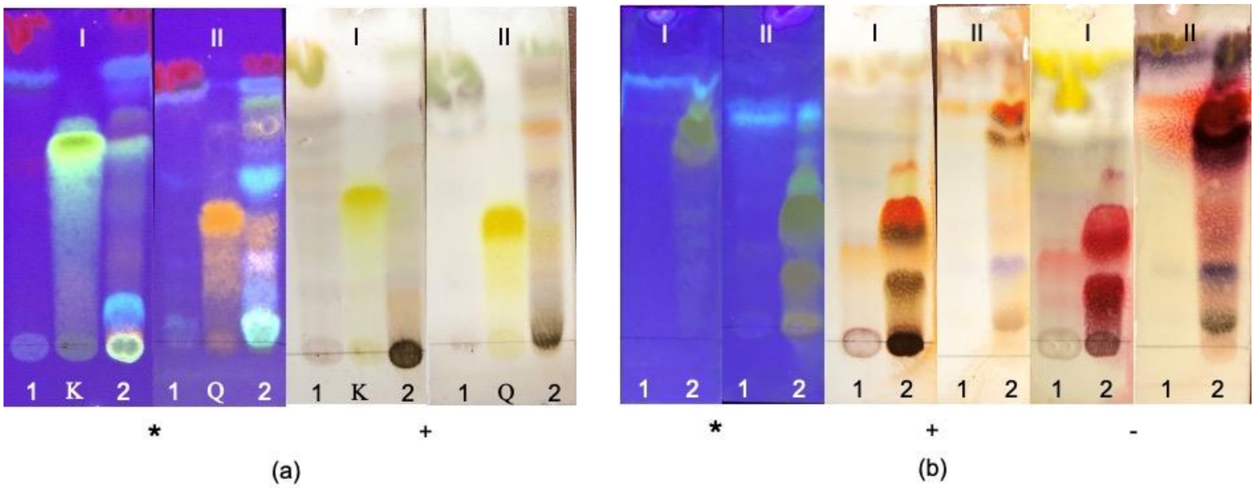

Figure S1: TLC chromatograms of four *S. chamaejasme* extracts obtained with DCM (1) and EtOH (2) from aerial parts (a) and roots (b) and two standards (K: kaempferol, Q: quercetin). In I and II, the mobile phases used were composed of chloroform and methanol (9:1, v/v) and chloroform, methanol and water (7:3:0.4, v/v/v), respectively. Chromatograms were sprayed with NP/PEG (*) and revealed using UV (365 nm) and sulfuric acid (+) or vanillin-sulfuric reagent (-) and evaluated in visible light.

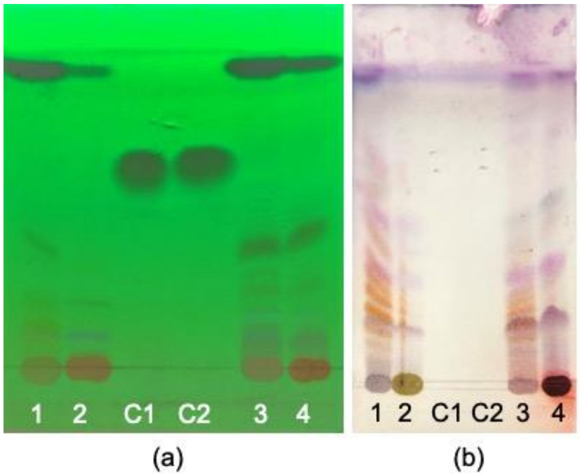

Figure S2: TLC chromatograms of *S. chamaejasme* extracts including, DCM-AP (1), EtOH-AP (2), DCM-R (3) and EtOH-R (4) and two standards (C1: coumarin from National University of Mongolia, C2: coumarin from MONOS pharmaceutical industry). The mobile phases were chloroform. Chromatogram was evaluated in UV 254 nm (a). Other chromatogram was sprayed with vanillin-sulfuric reagent (b) and evaluated in visible light.

SUPPLEMENTARY MATERIALS to “Antioxidant and anti-inflammatory activities of *Stellera chamaejasme* roots and aerial parts extracts” by Temuulen Selenge , Sara F. Vieira, Odontuya Gendaram, Rui L. Reis, Soninkhishig Tsolmon, Enkhtuul Tsendeekhuu, Helena Ferreira and Nuno M. Neves.

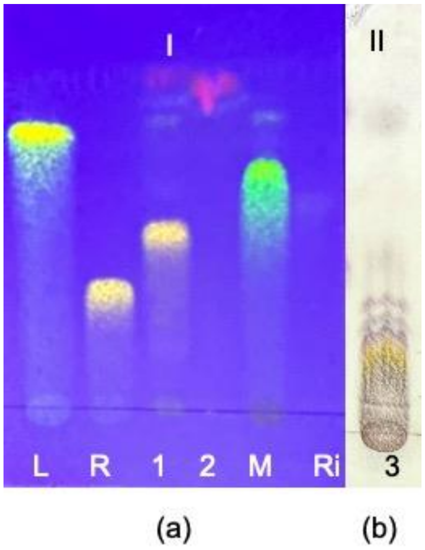

Figure S3: TLC chromatograms of two *S. chamaejasme* extracts including, EtOH-AP (1), DCM-AP (2), and DCM-R (3), and four standards (L: Luteolin, R: Rutin, M: Morin, Ri: Riboflavin). In I and II, the mobile phases used were composed of chloroform, methanol and water (7:3:0.4, v/v/v) and dichloromethane, respectively. Chromatograms were sprayed with NP/PEG (a) and revealed using UV (365 nm) or vanillin-sulfuric reagent (b) and evaluated in visible light.

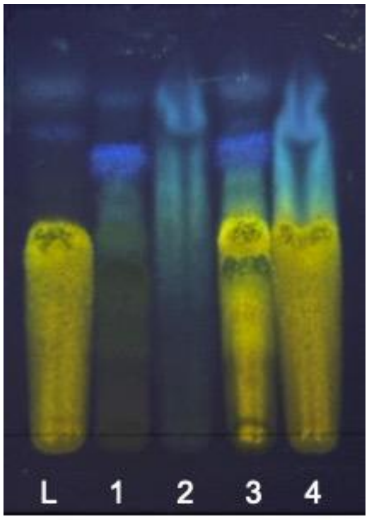

Figure S4: TLC chromatograms of *S. chamaejasme* extracts including, EtOH-R (1), DCM-R (2), EtOH-R+ Luteolin (3) and DCM-R + Luteolin (4) and the standards luteolin (L). The mobile phases used was composed of chloroform and methanol (6:1, v/v). Chromatogram was sprayed with NP/PEG and revealed using UV (365 nm).

SUPPLEMENTARY MATERIALS to "Antioxidant and anti-inflammatory activities of *Stellera chamaejasme* roots and aerial parts extracts" by Temuulen Selenge, Sara F. Vieira, Odontuya Gendaram, Rui L. Reis, Soninkhishig Tsolmon, Enkhtuul Tsendeekhuu, Helena Ferreira and Nuno M. Neves.

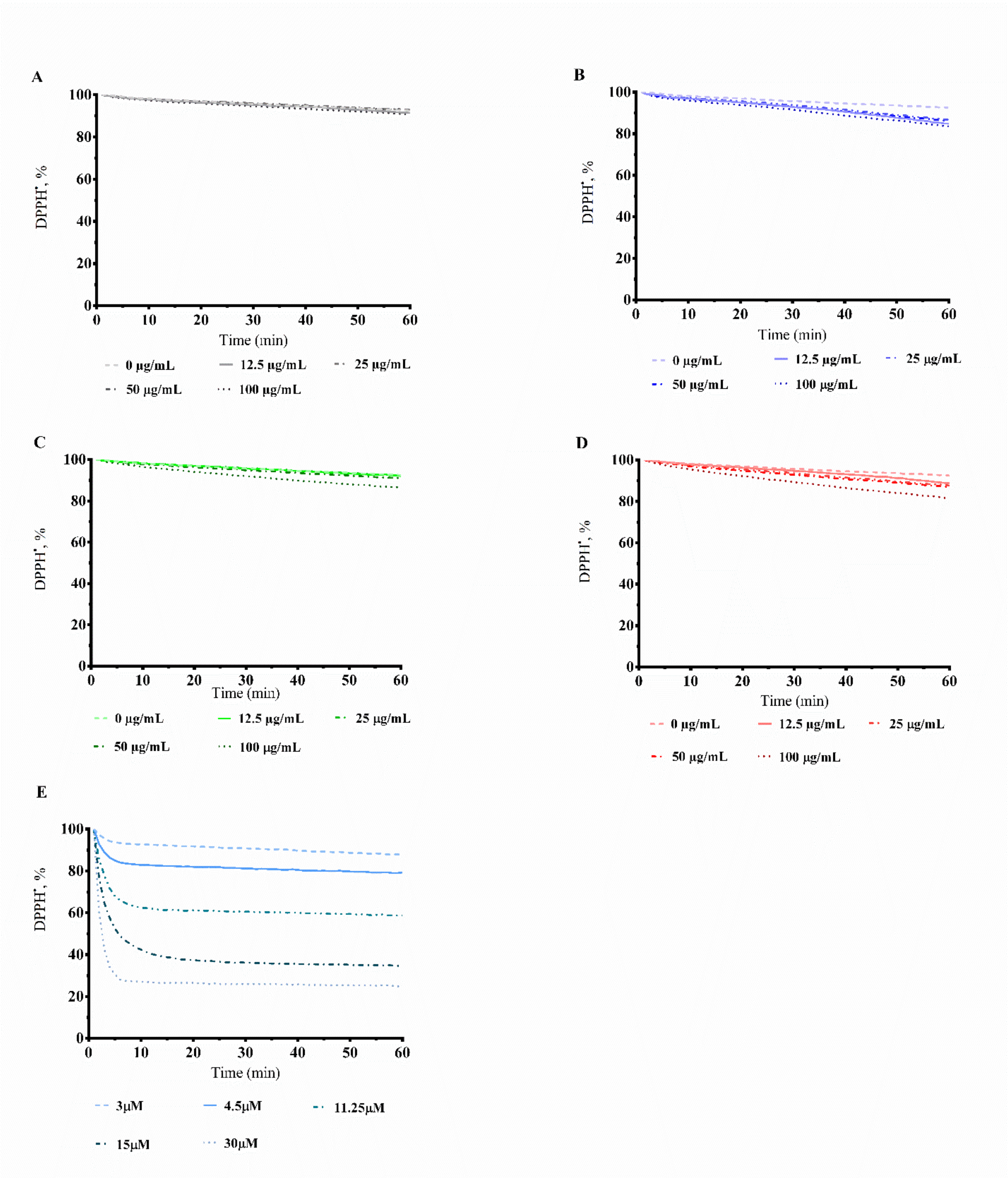

Figure S5: Antioxidant activity against DPPH• of varying concentrations of EtOH-AP (A), DCM-AP (B), EtOH-R (C), DCM-R (D) extracts and trolox (E).

SUPPLEMENTARY MATERIALS to “Antioxidant and anti-inflammatory activities of *Stellera chamaejasme* roots and aerial parts extracts” by Temuulen Selenge, Sara F. Vieira, Odontuya Gendaram, Rui L. Reis, Soninkhishig Tsolmon, Enkhtuul Tsendeekhuu, Helena Ferreira and Nuno M. Neves.

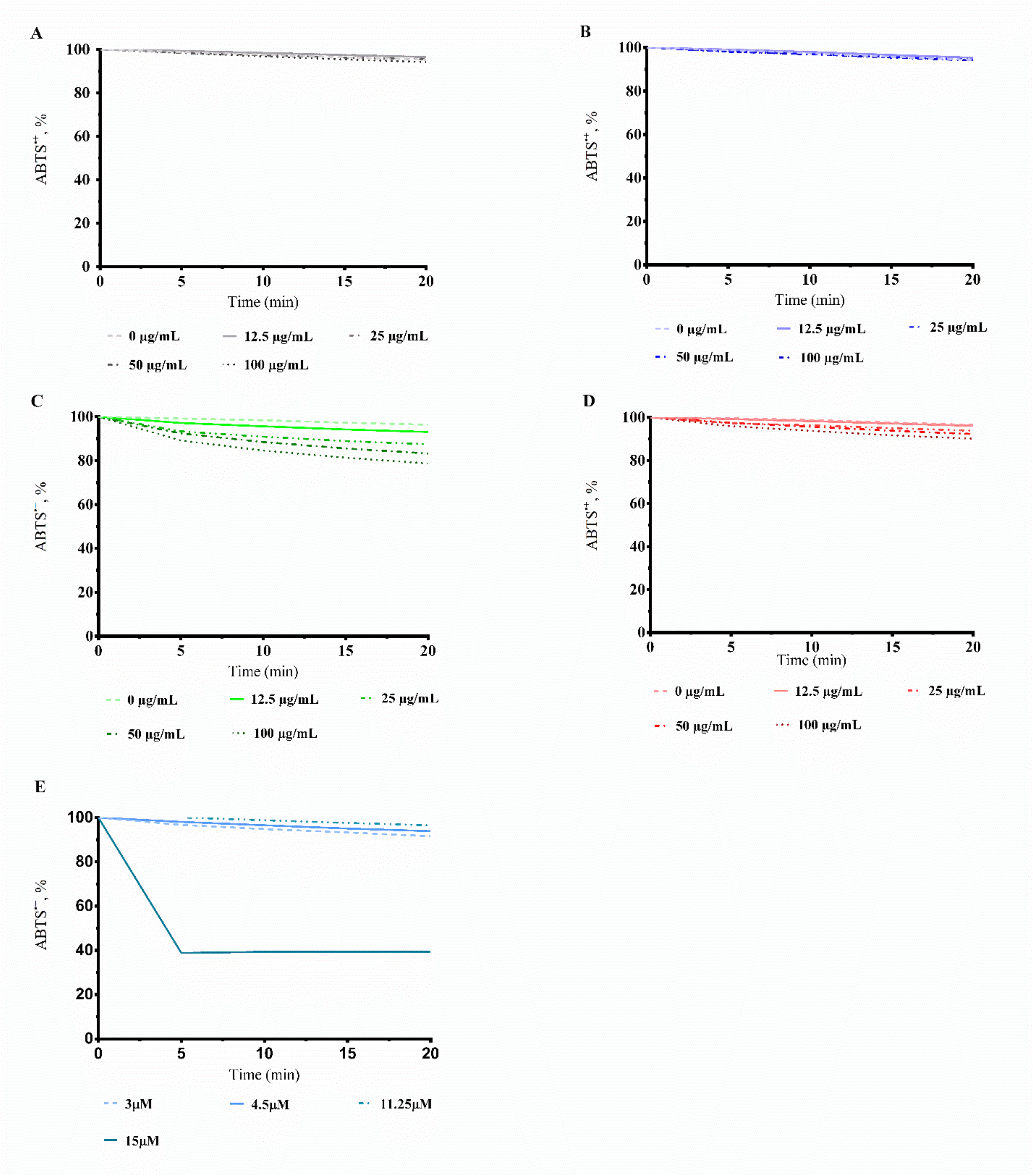

Figure S6: Antioxidant activity against ABTS$^{\cdot+}$ of varying concentrations of EtOH-AP (A), DCM-AP (B), EtOH-R (C), DCM-R (D) extracts and trolox (E).
